# Evolution of the relaxin-like peptide family

**DOI:** 10.1186/1471-2148-5-14

**Published:** 2005-02-12

**Authors:** Tracey N Wilkinson, Terence P Speed, Geoffrey W Tregear, Ross AD Bathgate

**Affiliations:** 1 Howard Florey Institute of Experimental Physiology and Medicine, University of Melbourne, Australia; 2 Walter and Eliza Hall Institute of Medical Research, Parkville, Victoria, Australia

## Abstract

**Background:**

The relaxin-like peptide family belongs in the insulin superfamily and consists of 7 peptides of high structural but low sequence similarity; relaxin-1, 2 and 3, and the insulin-like (INSL) peptides, INSL3, INSL4, INSL5 and INSL6. The functions of relaxin-3, INSL4, INSL5, INSL6 remain uncharacterised. The evolution of this family has been contentious; high sequence variability is seen between closely related species, while distantly related species show high similarity; an invertebrate relaxin sequence has been reported, while a relaxin gene has not been found in the avian and ruminant lineages.

**Results:**

Sequence similarity searches of genomic and EST data identified homologs of relaxin-like peptides in mammals, and non-mammalian vertebrates such as fish. Phylogenetic analysis was used to resolve the evolution of the family. Searches were unable to identify an invertebrate relaxin-like peptide. The published relaxin cDNA sequence in the tunicate, *Ciona intestinalis *was not present in the completed *C. intestinalis *genome. The newly discovered relaxin-3 is likely to be the ancestral relaxin. Multiple relaxin-3-like sequences are present in fugu fish (*Takifugu rubripes*) and zebrafish (*Danio rerio*), but these appear to be specific to the fish lineage. Possible relaxin-1 and INSL5 homologs were also identified in fish and frog species, placing their emergence prior to mammalia, earlier than previously believed. Furthermore, estimates of synonymous and nonsynonymous substitution rates (*d*_N_*/d*_S_) suggest that the emergence of relaxin-1, INSL4 and INSL6 during mammalia was driven by positive Darwinian selection, hence these peptides are likely to have novel and in the case of relaxin-1, which is still under positive selection in humans and the great apes, possibly still evolving functions. In contrast, relaxin-3 is constrained by strong purifying selection, demonstrating it must have a highly conserved function, supporting its hypothesized important neuropeptide role.

**Conclusions:**

We present a phylogeny describing the evolutionary history of the relaxin-like peptide family and show that positive selection has driven the evolution of the most recent members of the family.

## Background

The relaxin-like peptide family includes: relaxin-1, relaxin-2, relaxin-3, and the insulin-like (INSL) peptides, INSL3, INSL4, INSL5 and INSL6. All share high structural similarity with insulin due to the presence of six cysteine residues, which confer two inter-chain and one intra-chain disulfide bonds. Thus, it was postulated that relaxin and insulin had derived from a common ancestral gene and were therefore grouped as the insulin superfamily, which later included insulin-like growth factors I and II (IGF-1 and -2) (reviewed in [1]).

Despite less than 50% predicted sequence similarity across members of the insulin superfamily, primary structural determinants are retained, resulting in the similar tertiary structures of relaxin and insulin (reviewed in [1]). The structures of insulin, relaxin [1], relaxin-3 [2] and INSL3 [3] are formed by the cleavage of the pro-hormone peptide into three chains (A, B and C), removal of the C chain and the formation of three disulfide bridges between six invariant cysteine residues found on the A and B chains, to produce an active protein. Based on primary sequence similarity the native structures of human relaxin-1 (H1 relaxin), INSL4, 5 and 6 should be similar, but to date this has not been confirmed.

Various studies have highlighted the importance of a [Arg-X-X-X-Arg-X-X-Ile] motif in the relaxin B chain for interaction with the relaxin receptor and biological activity, which is dependent upon the presence of this motif [4]. Coupled with the insulin-like cysteine bond pattern, the presence of this motif is used to distinguish relaxin sequences. Hence, three relaxin genes are present in humans: relaxin-1 is found only in humans and the great apes, its expression is limited to the decidua, placenta and prostate [5]; relaxin-2 is the major circulating form of relaxin in the human [6] and the functional equivalent to the relaxin-1 in all non-primates; while relaxin-3 was only recently discovered and shows brain specific expression [2]. Throughout this paper relaxin will be used to refer to relaxin-2 in humans and great apes, and the equivalent relaxin-1 in all other mammals (table 1).

**Table 1:** The relaxin-2 gene in humans and great apes is the equivalent of the relaxin-1 gene in non-primate species and will be referred to as relaxin throughout this paper. Non-primate species only have *RLN1 *and *RLN3 *genes.

Humans and Great Apes	All other mammals e.g. mouse	Peptide abbreviation	Gene name
Relaxin-1	Relaxin^#^	RLX1	*RLN1*
Relaxin-2^#^		RLX2	*RLN2*
Relaxin-3	Relaxin-3	RLX3	*RLN3*

^#^indicates functional equivalence

Relaxin has been well characterized in a reproductive context.. It is a product of the ovary and/or placenta in most species studied, and has various roles in pregnancy and parturition, which differ between species [7]. Recent advances have revealed relaxin to be a multifunctional hormone, with numerous non-reproductive roles (reviewed in [1]). Of the other relaxin-like peptides, INSL3, or relaxin-like factor (RLF), is closely related to relaxin and is critical for testis descent [8,9]. Early placenta insulin-like peptide (EPIL), placentin or insulin-like 4 (*INSL4*) is primate specific and likely to have diverged from a common relaxin ancestor before the duplication that led to the two relaxin genes seen in humans and great apes [10]. Insulin-like 5 and 6 (*INSL5 *and *INSL6*) were identified by searching EST databases with the cysteine motif conserved in all insulin-like peptides [11,12]. Both INSL5 and 6 peptides show higher sequence similarity to relaxin rather than insulin and are functionally uncharacterised.

Until recently, receptors for all peptides of the relaxin family were unknown. Given the high degree of structural similarity between relaxin and insulin, it had been believed that the relaxin receptor would also be a tyrosine kinase receptor, similar to the insulin receptor. However, it was finally demonstrated in 2002, that relaxin activated two previously orphan leucine rich repeat containing heterotrimeric guanine nucleotide binding protein-coupled receptors (GPCR), LGR7 and LGR8 [13]. Further studies have shown that LGR7 is the relaxin receptor [13], although it also specifically interacts with relaxin-3 [14], and that LGR8 is the INSL3 receptor [15,16]. Even more recently, another two GPCRs which are activated by relaxin-3, the somatostatin- and angiotensin- like peptide receptor (SALPR or GPCR135) and GPCR142 have been identified [17,18]. More recently GPCR142 has been shown to be the receptor for INSL5, [19] while GPCR135 appears to be the specific receptor for relaxin-3. Surprisingly, while LGR7, LGR8, GPCR135 and GPCR142 are all Type I GPCRs, they are from different branches within this family and are only very distantly related [17,18,20]. No other relaxin-like peptides have been shown to interact with these receptors [13,14,16-18].

Relaxin and INSL3 have primarily been of interest as hormones of pregnancy and reproduction; therefore it was assumed that the largely uncharacterised INSL4, 5 and 6 would share similar functions. However the discovery of the brain specific relaxin-3, and the widespread expression of INSL5 and its receptor GPCR142 have resulted in a re-evaluation of these assumptions and raised the need for a new approach to investigating these peptides.

Relaxin evolution has confounded researchers for decades. High sequence variability in relaxins across closely related species is a well-known feature of this peptide, however startling similarities have been observed between very distant species such as pigs and whales [21]. Other studies have reported the existence of an invertebrate relaxin, a hormone with "relaxin-like" properties has been described in protozoa (*T. pyriformis*) [22], ascidians (*H. momus*) [23] and tunicates (*C. intestinalis*) [23]. A cDNA and peptide sequence with almost 100% similarity to porcine relaxin was isolated from *C. intestinalis *[24]. Contrastingly, numerous efforts to identify and sequence a relaxin ortholog in bovine and other ruminants have been unsuccessful. A likely non-functional single copy relaxin-like gene has been found in the ovine [25]. Despite this controversy very few phylogenetic studies of relaxin, or the other relaxin-like peptides, have been reported. An in-depth analysis analysing only relaxin-1, -2 and INSL3 in several primates has been performed [26] and a more recent paper has discussed the evolution of the family without including a detailed phylogenetic analysis [27].

The increasing availability of genomic data has provided an opportunity to clarify the area of relaxin evolution using phylogenetic analysis. The relaxin-like peptide family phylogeny shows relaxin-3 is likely to be the ancestral relaxin, emerging prior to the divergence of fish, presumably with a function in the brain. Multiple relaxin-3 sequences are present in the fish and frogs, while the phylogeny also suggests that a relaxin with reproductive functions is present in these lineages. However, relaxin has been lost in the chicken and its genome instead contains two relaxin-3-like sequences. Evolutionary rate analysis shows positive Darwinian selection to be driving the most recent members of the family, INSL6, INSL4 and relaxin-1.

## Results

### Sequence similarity searches and multiple alignment

Table 2 outlines the relaxin-like peptide sequences used in these phylogenetic analyses, from human, mouse, rat, dog, chimpanzee, pig, chicken, wallaby, *R. esculenta*, *X. tropicalis*, *X. laevis*, fugu fish, zebrafish, rhesus monkey and rainbow trout. New sequences identified during similarity searches have been highlighted. The source of each newly identified sequence is annotated as a footnote (i.e. genomic, ESTs). It should be noted that more mammalian relaxin and INSL3 sequences have been identified than were included in these analyses. Inclusion of these sequences did not improve the accuracy of the phylogeny, and as the aim of this study was to determine the evolutionary history of the entire family, they were omitted. All available non-mammalian relaxin-like sequences with accompanying nucleotide sequences were included.

**Table 2:** GenBank accession numbers for relaxin-like sequences. Accession numbers for all sequences included in the phylogeny are listed. New sequences identified in this study are highlighted in bold and accession numbers shown in brackets underneath. Phylogenetic analyses showed TrRLX3f, DrRLX3c, XtRLX3a and XlRLX3 sequences (shown in italics) to be relaxin rather than relaxin-3 homologs and are therefore listed in the relaxin (RLX2) column.

Species	RLX1	RLX2	RLX3	INSL3	INSL4	INSL5	INSL6
Human	P04808	P04090	Q8WXF3	P51460	Q14641	Q9Y5Q6	Q9Y581
Chimpanzee	S42783	P51455	**BK005156^b^**	**BK005155^b^**	**BK005152^b^**	**BK005153^b^**	**BK005154^b^**
Mouse	-	CAA81611	Q8CHK2	O09107	-	Q9WUG6	Q9QY05
Rat	-	J00780	Q8BFS3	AAD33663	-	-	Q9WV41
Dog	-	Q9TRM8	AAEX01023146	AAEX01022997			AAEX01053723
Wallaby		AAM22209					
*R. esculenta*		CAC16108					
Fugufish		***RLX3f*** *BK005388*^*b*^	RLX3a-c			RLX3d,e	
Zebrafish		***RLX3c*** *BK005255*^*b*^	RLX3a BK005228			**RLX3b** BK005252^a,b^ **RLX3d **(^b,c^)	
Chicken			**RLX3a** BK005146^a^			**RLX3b** BK005533^b^	
Pig		P01348	Q8HY17	P51461			
*X. tropicalis*		***RLX3a*** *BK005229*^*a*^	**RLX3b**(^b,d^) **RLX3c**(^b,d^)				
*X. laevis*		***RLX3*** *BK005230*^*a*^					
*O. mykiss*						**RLX3** BK005147^a^	
*M. mulatta*					**INSL4** BK005251^a^		

^a^Sequences determined from multiple overlapping ESTs.

^b^Sequences determined from genomic data.

^c^Sequence from the Ensembl zebrafish genome assembly [68](GENSCAN00000013529 on supercontig NA8544).

^d^Sequence from the Xenopus genome v2.0 [58].

Searches of the completed human, mouse and rat genomes did not identify any novel relaxin-like peptides, however, likely non-functional INSL5 genes were revealed in the rat and dog genomes. A sequence with high similarity to INSL5 was found (Genbank Accession No: NW_047717.1), with a frameshift mutation in the first exon of the gene, which introduces a stop codon, resulting in a protein truncated early in the B chain (data not shown). The recently completed dog genome also contains a sequence highly homologous to the human INSL5 peptide (Genbank Accession No: NW_ AAEX01024390.1). However this sequence does not encode an open reading frame, with two stop codons present in the C peptide sequence and one upstream of a homologous B chain sequence. Furthermore, there was no recognizable signal peptide or Methionine start codon upstream of this B chain sequence and it was missing the critical Cys-29 in the B chain.

**Figure 1:**
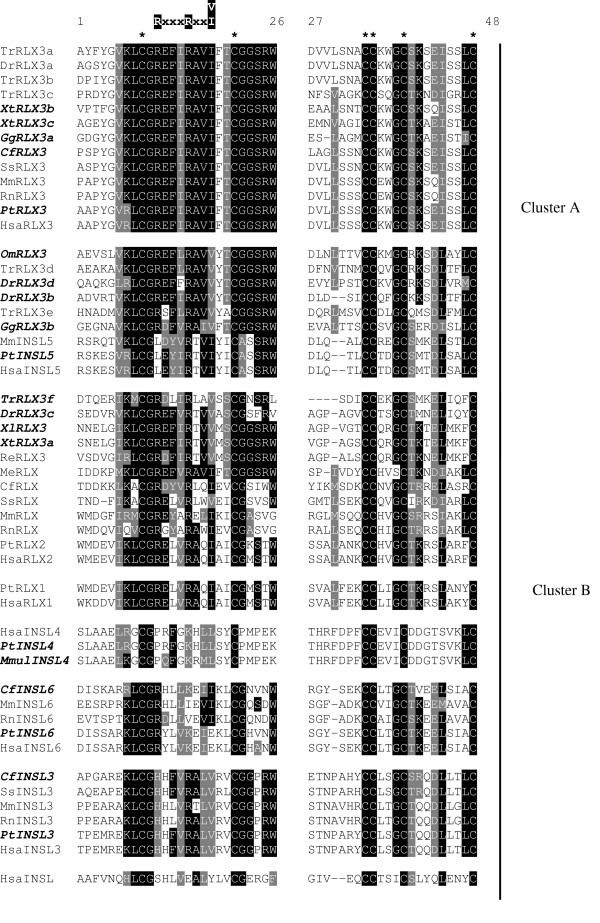
**Multiple sequence alignment of the relaxin-like peptide family. **Amino acid sequences of the B and A domains only were aligned using ClustalW, then edited by hand using Seaview to remove gaps. This alignment was then used for all phylogenetic analyses. Newly identified sequences are highlighted in bold and italics. Invariant cysteine residues are indicated by asterisks (*) and the relaxin specific B-chain motif [RxxxRxxI/V] is shown. Sequences are clustered into subfamilies (A and B) based on primary sequence similarity and phylogenetic analysis. Hsa = *Homo sapiens*, Pt = *Pan troglodytes*, Mmul = *Maca mulatta*, Mm = *Mus musculus*, Rn = *Rattus norvegicus*, Cf = *Canis familiaris*, Ss= *Sus scrofa*, Re = *Rana esculenta*, Me = *Macropus eugenii*, Xl = *Xenopus laevis*, Xt = *Xenopus tropicalis*, Dr = *Danio rerio*, Tr = *Takifugu rubripes*, Gg = *Gallus gallus*, Om = *Oncorhynchus mykiss*.

Orthologs of all relaxin-like peptides were identified in the completed chimpanzee genome (table 2). Two relaxin-like sequences were identified in the completed chicken genome (GgRLX3a, b in table 2 and figure 1), both with high similarity to human relaxin-3. Comparing the putative B and A domains of GgRLX3a and b with those of human relaxin-3 shows them to be 81% and 75% similar respectively (data not shown). Three sequences with a high similarity to human relaxin-3 (73%, 92% and 90% respectively; data not shown) were also discovered in the *X. tropicalis *genome (XtRLX3a-c, in table 2 and figure 1).

Sequences with high similarity to relaxin-3 have previously been reported in the fugu fish, TrRLX3a-e [27] and zebrafish, DrRLX3a [27,28] (see table 2 and figure 1). These searches identified a sixth relaxin-like sequence in the fugu fish, TrRLX3f, and another three in the zebrafish, DrRLX3b-d (table 2 and figure 1). Unlike the sequences previously identified in the fugu fish [27], TrRLX3f is more similar to human relaxin-2 (60%) than human relaxin-3 (54%) (Data not shown). Of the zebrafish sequences DrRLX3b and d both show 77% similarity to human relaxin-3 in their B and A domains (data not shown). DrRLX3c is only 60% similar to human relaxin-3 and 54% similar to human relaxin-2 (data not shown).

Searches of partially completed genomes identified relaxin-like sequences in *X. laevis *(XlRLX3), *Oncorhynchus mykiss *(rainbow trout) (OmRLX3) and an INSL4 ortholog in the rhesus monkey (table 2 and figure 1). While OmRLX3 shows high similarity to human relaxin-3 (76%, data not shown), XlRLX3 is less similar (69%, data not shown).

The presence of a relaxin gene in ruminants could not be determined due to the incomplete bovine genomic data currently available. No bovine ESTs with a similarity to relaxin were found and the presence of a bovine equivalent to the ovine likely non-functional genomic relaxin sequence could not be confirmed. However, a bovine EST was identified (BI682322) with high similarity to human relaxin-3 (79% identity to the translated EST product) starting from the end of the B chain (45F in the human pro-hormone sequence).

Searches of invertebrate genomic and EST databases failed to identify a relaxin-like gene in any invertebrate or prokaryote. Although a *C. intestinalis *relaxin-like cDNA sequence has previously been reported [24], our searches failed to confirm this finding. The published relaxin sequence could not be found in the completed *C. intestinalis *genome.

The B and A chains from all relaxin-like peptides identified in the following species: human, chimpanzee, rhesus monkey, pig, mouse, rat, wallaby, chicken, fugu fish, zebrafish, rainbow trout,*R. esculenta*, *X. laevis *and *X. tropicalis *were aligned using ClustalW and edited to remove all gaps, which are problematic in phylogenetic analysis (figure 1). Only the six cysteine residues responsible for conferring structure are conserved across all the relaxin-like peptides. Striking identity is seen amongst relaxin-3 sequences, especially in the B chain. Much lower similarity is seen amongst relaxin sequences, apart from the cysteine motif, only the relaxin-specific B chain motif is conserved. C peptide sequences show only negligible similarity between even closely related species making them impossible to align accurately. The C peptide is cleaved from the mature form of relaxin-1, 3 and INSL3, is believed to be cleaved from the mature form of all other relaxin-like peptides and was therefore excluded from all sequences.

### Phylogeny of the relaxin-like peptide family

The alignment of B and A domains described above (figure 1) was used to construct phylogenetic trees with the maximum parsimony (MP), neighbour-joining (NJ) and maximum likelihood (ML) methods. MP and NJ methods produced conflicting trees, both with low bootstrap support for most of the major branches, and the ML tree failed to resolve many relationships (data not shown). Based on the relationships that could be determined with a degree of confidence, the sequences were divided into two clusters to be analysed separately (as shown in figure 1). Cluster A contained relaxin-3 and INSL5 sequences, while cluster B contained relaxin-1, relaxin-2, INSL3, INSL4 and INSL6 sequences. Several fish and frog sequences with lower sequence similarity to relaxin-3 (DrRLX3c, TrRLX3f, XlRLX3 and XtRLX3, figure 1) were grouped with cluster B based on preliminary phylogenetic analysis (data not shown). These sequences have, therefore, been listed as relaxin homologs rather than relaxin 3 (table 2). Also placed in cluster B are the sequences previously isolated from the tammar wallaby, MeRLX, [29] and edible frog, ReRLX [30]. Despite sequence similarity to relaxin-3, previous functional characterization and expression profiles of MeRLX and ReRLX indicates they are relaxin, rather than relaxin-3 homologs.

**Figure 2:**
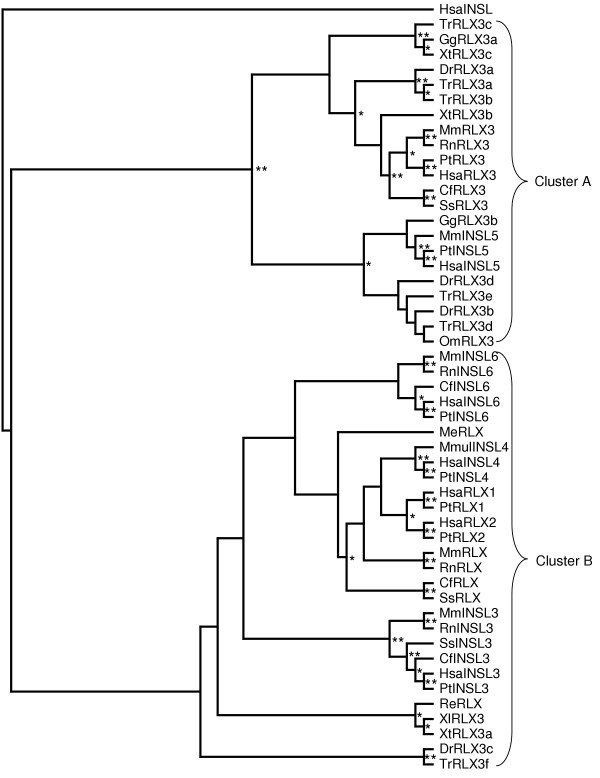
**Evolutionary relationships among relaxin-like peptides. **Topology shown is a consensus tree based on MP (maximum parsimony), ML (maximum likelihood) and NJ (neighbour-joining) analysis of the amino acid alignment shown in figure 1. Consensus tree was produced and edited using TreeView to correlate topology with known genomic information about the family. Human insulin used as an outgroup. Where possible, confidence values are shown at branches: * >50%, ** >75%, all other branches are inferred. Hsa = *Homo sapiens*, Pt = *Pan troglodytes*, Mmul = *Maca mulatta*, Mm = *Mus musculus*, Rn = *Rattus norvegicus*, Cf = *Canis familiaris*, Ss = *Sus scrofa*, Re = *Rana esculenta*, Me = *Macropus eugenii*, Xl = *Xenopus laevis*, Xt = *Xenopus tropicalis*, Dr = *Danio rerio*, Tr = *Takifugu rubripes*, Gg = *Gallus gallus*, Om = *Oncorhynchus mykiss*.

**Figure 3:**
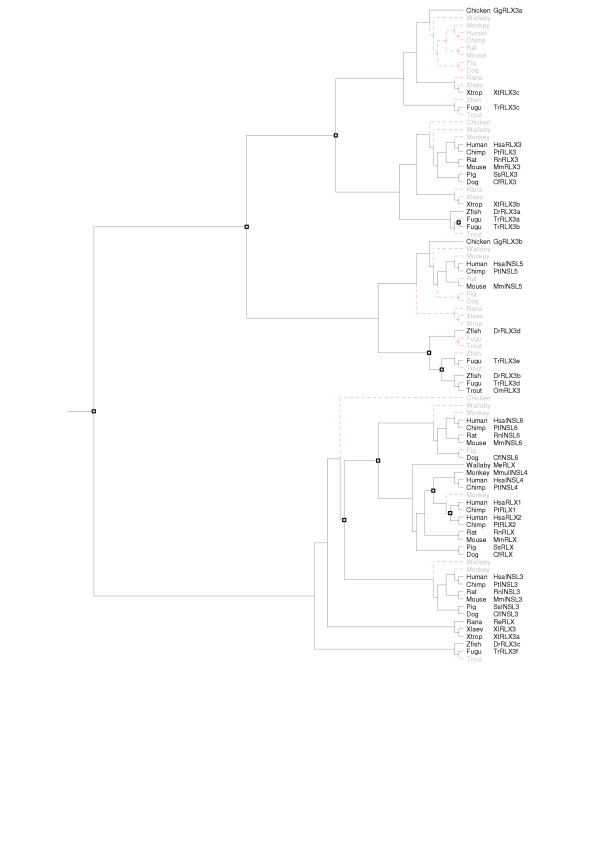
**Reconciled tree for the relaxin-like peptide family. **The consensus tree of relaxin-like peptides (figure 2) from human, chimpanzee, mouse, dog, rat, pig, wallaby, chicken, fugu fish, zebrafish, rainbow trout, *R. esculenta*, *X. laevis *and *X. tropicalis *was reconciled using GeneTree with a species tree complied from a phylogeny of model organisms [65]. Squares indicate duplication events, red dotted lines indicate absent genes, either lost from those species (in grey), or not yet sequenced. While used to construct the gene tree as an outgroup, insulin has been removed from the reconciled tree. Hsa = *Homo sapiens*, Pt = *Pan troglodytes*, Mmul = *Maca mulatta*, Mm = *Mus musculus*, Rn = *Rattus norvegicus*, Cf = *Canis familiaris*, Ss = *Sus scrofa*, Re = *Rana esculenta*, Me = *Macropus eugenii*, Xl = *Xenopus laevis*, Xt = *Xenopus tropicalis*, Dr = *Danio rerio*, Tr = *Takifugu rubripes*, Gg = *Gallus gallus*, Om = *Oncorhynchus mykiss*.

None of the tree construction methods employed was able to completely resolve the phylogeny of either cluster. Bootstrap values in the MP and NJ generated trees were very low (below 50%, data not shown) and Tree-Puzzle failed to resolve the position of several sequences. This is primarily because of the short sequences used, as the C peptide can not be used to increase sequence length and thus improve the output from the tree generation methods, an inferred tree was produced instead. The Tree-Puzzle tree was resolved using topologies conserved between the MP and NJ trees and then reconciled against a species tree using GeneTree. The inferred gene trees were then edited to minimize the incongruence (the number of losses and inferred duplications) with the species tree. The inferred cluster A Additional file 1  and the cluster B trees Additional file 2  were combined to produce the phylogenetic tree of the complete relaxin-like peptide family (figure 2). Branch confidence levels are indicated on figure 2; branches without notation are inferred only. This gene tree was then reconciled with the species tree (figure 3).

Analysis of the reconciled tree shows a major duplication event occurred early in the vertebrate lineage, giving rise to two subfamilies (clusters A and B respectively). Another duplication in subfamily A, prior to the emergence of fish, resulted in two lineages, which evolved into relaxin-3 and INSL5 in mammals. Interestingly, several non-mammalian relaxin-3-like sequences grouped with INSL5, implicating them as possible INSL5 homologs (GgRLX3b, OmRLX3, DrRLX3b, DrRLX3d, TrRLX3d and TrRLX3e). The reconciled tree also shows two additional fish-specific duplications in subfamily A. In the fugu fish genome a third duplication has occurred, resulting in three putative relaxin-3 (TrRLX3a, b, c) and two INSL5 homologs (TrRLX3d, e). In subfamily B there were four duplications, all were after the divergence of birds and reptiles and likely to have occurred during mammalian evolution. These events resulted in INSL3, INSL6, relaxin-1, relaxin-2 and INSL4.

### Synonymous (*d*_S_) and Nonsynonymous (*d*_N_) substitution rate estimates

Results show the relaxin-like peptides are under varying selection pressures (table 3). Pairwise comparisons of human and chimpanzee orthologs provide the only way to compare all members of the family between two species. *RLN1*, *RLN2 *and *INSL6 *have high *d*_N_*/d*_S _rate estimates, with results for *RLN1 *and *INSL6 *suggesting positive Darwinian selection. The extremely high estimate for *INSL6 *(99) is caused by having a *d*_S _of 0 (i.e. no synonymous substitutions), resulting in a division by 0 for the rate estimate, which is represented as 99 rather than infinity. All other human and chimpanzee sequences compared were identical and thus produced *d*_N_*/d*_S  _estimates of 0.

**Table 3:** Synonymous (*d*_S_) and nonsynonymous (*d*_N_) substitution rate estimates for all relaxin-like genes. Substitution rates were estimated using the Yang and Neilsen, 2000 method as implemented in yn00 in the PAML suite. Estimations were made using pairwise alignments of the nucleotide sequences of the B and A domains of human and chimpanzee or human and mouse genes. A *d*_N_/*d*_S _of 99 represents infinity and indicates that all substitutions detected are nonsynonymous, while na indicates that a *d*_N_/*d*_S _measurement is not possible as the sequences being compared are identical, or have only synonymous substitutions.

	human- chimpanzee			human-mouse		
	
	d_N_	d_S_	d_N_/d_S_	d_N_	d_S_	d_N_/d_S_
***RLN1***	0.055	0.039	1.4			_^a^
***RLN2***	0.017	0.023	0.7	0.56	1.06	0.5
***RLN3***	0	0	na	0.04	1.83	0.02
***INSL3***	0	0	na	0.17	1.31	0.1
***INSL4***	0	0.027	na			_^a^
***INSL5***	0	0.023	na	0.16	1.24	0.1
***INSL6***	0.006	0	99	0.33	0.65	0.5
***INS***	0	0.13	na	0.033	1.44	0.02

^a ^These genes are not present in the mouse.

Human and mouse orthologs were used to estimate rates for the other members of the family. *RLN2 *and *INSL6 *show the highest estimates again, although are much lower than comparisons with chimpanzee sequences, the *INSL6 *estimate suggesting weak purifying selection instead of positive selection. The very low substitution rate observed for *RLN3 *(0.02) shows this peptide to be under strong purifying selection, at a similar rate to insulin (*INS*) (table 3). Rates vary among the other members of the family from ~0.1 for *INSL3 *and *INSL5 *to ~0.5 for *RLN2 *and *INSL6*. As *INSL4 *is not present in mice and the human and chimpanzee sequences were identical, the *INSL4 *sequence from the rhesus monkey was used instead (data not shown). This comparison yielded a *d*_N_*/d*_S _estimate of 0.5, indicating weak purifying selection.

**Figure 4:**
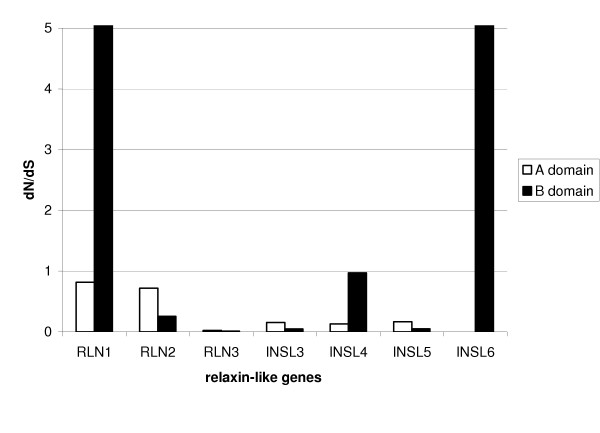
**Synonymous (*d*_S_) and nonsynonymous (*d*_N_) substitution rate estimates for individual B and A domains of each relaxin-like gene. **Substitution rates (*d*_N_/*d*_S_) were estimated using the Yang and Neilsen, 2000 method as implemented in yn00 in the PAML suite. Human and chimpanzee comparisons were used for RLN1 and INSL6; human and rhesus monkey comparisons were used for INSL4 and human, mouse comparisons were used for RLN2, RLN3, INSL3 and INSL5.

Substitution rate estimates for the individual B and A domains were determined in a similar fashion, using human and chimpanzee comparisons for *RLN1*, *RLN2 *and *INSL6*; human, rhesus monkey comparisons were used for *INSL4 *and human, mouse comparisons were used for *RLN3*, *INSL3 *and *INSL5 *(figure 4). These comparisons show the B domains of relaxin-1 and INSL6 to be under positive selection (both estimates were 99). The B domains of INSL4 and relaxin-2 also have high substitution rates (1.0 and 0.7 respectively), but are not high enough to suggest positive rather than neutral or weak purifying selection. All A domains are under the effects of fairly strong purifying selection, except for that of relaxin-1, which is under only very weak selection pressures (0.8). Interestingly, while the B domains of relaxin-2, INSL6 and INSL4 all have very high *d*_N_*/d*_S _estimates, the A domains of these genes have very low estimates. This is in contrast with the other members of the family, relaxin-3, INSL3 and INSL5, which all have higher *d*_N_*/d*_S  _estimates in the A domain than the B domain.

### Positive selection tests

**Figure 5:**
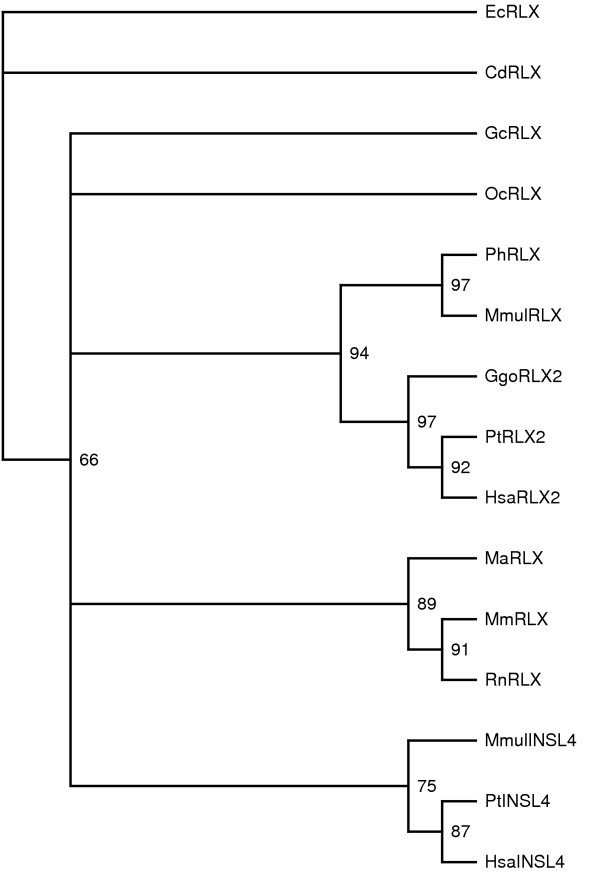
**Phylogeny of mammalian *RLN2 *and *INSL4 *genes. **Tree generated using Tree-Puzzle using a gamma distribution, the Dayhoff model of substitution and 10 000 puzzling steps. Confidence values are shown as percentages on each branch. The INSL4 branch (labeled A on the tree) was tested for positive selection. Hsa = *Homo sapiens*, Mm = *Mus musculus*, Rn = *Rattus norvegicus*, Ss = *Sus scrofa*, Me = *Macropus eugenii*, Mmul = *Maca mulatta*, Cd = *Camelus dromedaries*, Gc = *Galago crassicaudatus*, Pt = *Pan troglodytes*, Ggo = *Gorilla gorilla*, Ph = *Papio hamadryas*, Fc = *Felis catus*, Cf = *Canis familiaris*, Oc = *Oryctolagus cuniculus*, Ma = *Mesocricetus auratus*, Ec = *Equus caballus*.

To confirm the pairwise *d*_N_*/d*_S _(or ω) estimates, more sophisticated codon-based substitution models (reviewed in [31]) were used. As pairwise comparisons have already shown positive selection to be acting on *RLN1 *and *INSL6*, *INSL4 *was analysed further. The phylogenetic tree of all sequences used is shown in figure 5. Branch-specific likelihood analysis of the data, which assumes a constant ω ratio across all sites in a sequence, was used to test whether the INSL4 branch (branch A, figure 5) has a different ω ratio than all other branches. While the two-ratios model indicates a ω ratio of 1.1 for branch A (table 4), the LRT comparing this with the one-ratio model shows this to be statistically insignificant (*P *= 0.6, d.f. = 1, table 5).

**Table 4:** Parameter estimates for INSL4 under different branch models, site models and branch-site models. Models implemented in Codeml from the PAML suite. Parameters in boldface indicate positive selection. Sites potentially under positive selection are numbered using the human INSL4 sequence in figure 1 as the reference.

**Model**	**ρ**	**ℓ**	**Parameter est.**	**Positively selected sites**
1 ratio (R0)	1	-1460.1	ω = 0.7	

Branch specific				

2 ratios (R2)	2	-1459.9	ω_0 _= 0.7(background), **ω_1 _= 1.1 **(branch A)	

Site specific				

Neutral (M1)	1	-1424.4	ρ_0 _= 0.1, ρ_1 _= 0.8	not allowed
Selection (M2)	3	-1416.6	ρ_0 _= 0.1, ρ_1 _= 0.6, **ρ_2 _= 0.2, ω_2 _= 2.9**	15L (*P *>0.99) 14H, 27H, 28R, 36V (*P *>0.95)
Discrete (M3) (k = 2)	3	-1421.4	ρ_0 _= 0.2, ω_0 _= 0.03, **ρ_1 _= 0.8, ω_1 _= 1.0**	37 sites^a ^(*P *>0.99)
Discrete (M3) (k = 3)	5	-1413.8	ρ_0 _= 0.1, ω_0 _= 0.01, ρ_1 _= 0.5, ω_1 _= 0.6, **ρ_2 _= 0.3, ω_2 _= 2.0**	15L, 27H, 28R (*P *>0.99) 14H, 25G, 26R, 30D, 36V (*P *> 0.90)
Beta (M7)	2	-1419.6	ρ_0 _= 0.2,q = 0.2	not allowed
Beta&ω (M8)	4	-1414.6	ρ_0 _= 0.8,p = 0.3, q = 0.06, **ρ_1 _= 0.2, ω_1 _= 2.6**	15L, 28R (*P *> 0.95) 14H, 27H, 36V (*P *> 0.90)

Branch-Site				

Model A	3	-1418.4	ρ_0 _= 0.1, ρ_1 _= 0.6, **ρ_2 _= 0.2, ω_2 _= 3.0**	In the foreground lineage: 13K, 37I (*P *> 0.95)
Model B	5	-1416.4	ρ_0 _= 0.1, ω_0 _= 0.01, ρ_1 _= 0.6, ω_1 _= 0.6, **ρ_2 _= 0.3, ω_2 _= 3.2**	In the foreground lineage: 13K, 37I (*P *> 0.95) In the background lineages: no significant sites

^a^1A, 2A, 3E, 5R, 6G, 10R, 11F, 12G, 14H, 15L, 16L, 17S, 18Y, 20P, 25G, 26R, 27H, 28R, 29F, 30D, 31P, 32F, 35E, 36V, 37I, 39D, 40D, 41G, 42T, 43S, 44V, 45K, 47L. Note that these sites should be treated with caution as ω under this model is not significantly higher than 1.

**Table 5:** Likelihood ratio test statistics (2δ) for the INSL4 data set.

	2δ	d.f.	*P*-value
LRT of ω at branch A			
1 ratio (R0) vs. 2 ratios (R2)	0.3	1	0.6

**LRTs of variable ω's among sites**			

M0 vs. M3 (k = 3)	92.5	2	<0.0001
M1 vs. M2	15.7	2	0.0004
M7 vs. M8	10.1	2	0.006

**LRT's of variable ω's along branch A**			

M1 vs. Model A	12.0	2	0.002
M3 (k = 2) vs. Model B	10.0	2	0.007

Site-specific models, which allow the ω ratios to vary between sites in a sequence, were also applied to the data. The ω ratio was found to vary considerably among amino acid sites. The discrete model with *K *= 3 site classes was the best fit to the data with a log likelihood value of -1413.8 (table 4). This model suggests that 31% of sites are under positive selection (ω_2 _= 2.00), while half (54%) are under weak purifying selection (ω_1 _= 0.6) and the other 15% constrained under extreme purifying selection (ω_0 _= 0.01) (listed in table 4). Eight amino acids are identified as under positive selection at the 90% cut off (14H, 15L, 25G, 26R, 27H, 28R, 30D, 36V, see table 4). All but two of these are within the A chain of *INSL4*. The LRT of M3 with its null model (M0) shows these results to be significant (*P *< 0.0001, d.f. = 2, table 5). Similar results are seen with Model M8.

Lastly, branch-site models A and B were applied to the data. These models extend the site and branch specific models by allowing the ω ratios to vary among lineages and sites and were used to test for specific sites under positive selection on the *INSL4 *branch. Model A, which fits the data significantly better than its null model M0 (*P *= 0.02, d.f. = 2, table 5) identifies 2 sites (13K and 37I) under positive selection in the *INSL4 *branch at the 95% cut off (table 4). Model B, which allows the ω ratio to vary both in the foreground lineage (the *INSL4 *branch) and in the background branches also fits the data significantly better than its null model, M3 with k = 2 (*P *= 0.007, d.f. = 2, table 5), and identifies the same two positively selected sites as Model A (13K and 37I) in the *INSL4 *branch (table 4). Model B also confirms the results produced by the discrete model (M3 with k = 3 site classes), showing 27% of sites of under positive selection, 58% under weak purifying selection (ω_2 _= 0.58) and 14% under very strong purifying selection (table 4).

## Discussion

While relaxin evolution has been the centre of much controversy (relaxin is often cited as a gene that conflicts with the Darwinian theory of evolution [24,32-34]), this report is the first attempt to describe the evolutionary history of the whole relaxin-like peptide family from a phylogenetic perspective. Previous studies have concentrated on the primate relaxins and relaxin-like factors [26], or not included detailed phylogenetic analyses [27]. We have sought to expand upon these by incorporating sequences identified in all the available completed genomes with a subset of cloned relaxin-like sequences, particularly those from non-mammalian species.

None of the phylogenetic tree construction programs used was able to completely resolve the evolution of the relaxin-like peptide family. This is likely due to variable divergence across the family and the short sequence length [35]. Incorporating results from the MP and NJ methods suggested positions for several branches that were unresolved after ML analysis. Minimizing incongruence between the gene and species trees by reducing the number of assumed duplications in the reconciled tree also provided a method to infer the evolutionary history of this family.

Similarly to previously published results, searches of available genomic and EST data failed to identify any novel members of the relaxin-like peptide family [28]. Given the stringent and well-described insulin family signature that revolves around the invariant cysteine residues that confer the insulin-like structure seen across the superfamily, we find it improbable that any novel relaxin or insulin-like sequences will be identified.

The presence of an invertebrate relaxin has been of speculation since 1983 when relaxin-like activity was first detected in the protozoa, *T. pyriformis *[22]. Similar activity was reported in *H. momus *[23] and in *C. intestinalis*, where a cDNA sequence almost identical to pig relaxin was found [24]. However, our searches of all completed invertebrate genomes (including *C. intestinalis*) failed to identify any relaxin-like sequences, including the published sequence. Multiple insulin-like peptides have been found in several invertebrates, including: *Bombyxi mori *(silkworm) [36], *D. melanogaster *[37] and *C. elegans *[38]. As these sequences lack the relaxin-specific motif, and show no homology to other relaxin family peptides, they are not considered part of the relaxin subfamily and therefore have not been included in these analyses. Much of the controversy surrounding relaxin evolution concerns the identification of an invertebrate relaxin sequence (a cDNA sequence from *Ciona intestinalis*) almost identical to pig relaxin (Georges and Schwabe, 1999). Completion of the *C. intestinalis *and other invertebrate genomes has allowed us to conclude that there is not a relaxin-like sequence in any invertebrate sequenced to date. If an invertebrate relaxin does exist, it does not contain the relaxin-specific motif characterized in vertebrates.

A hallmark of relaxin sequences is their high variability, even amongst closely related species. Relaxin-like peptide sequences isolated from two whales are almost identical to porcine relaxin [21], however as these sequences were derived from amino acid sequencing, without nucleotide or and genomic sequence available, they have not been able to be included in these phylogenetic analyses.

The presence of a functional relaxin in the ruminant lineage has yet to be confirmed [25]. More genomic data is required to confirm the presence of a non-functional relaxin gene sequence in the bovine, similar to that observed in the ovine [25]. Searches of the preliminary bovine genome assembly have failed to find a relaxin gene. Interestingly, a relaxin sequence has been identified in the camel [39] and relaxin expression found in the closely related llama and alpaca [40]. While classified as a ruminant, Camelidae have a unique reproductive anatomy and physiology [41]. A bovine EST (BI682322) with high similarity to exon 2 of human relaxin-3 was identified. Confirmation of the presence of relaxin and relaxin-3 orthologs in ruminants awaits further sequencing of the bovine and ovine genomes.

The presence of an avian relaxin has also been of speculation. While relaxin-like activity has been reported in the chicken [42], an avian relaxin-like peptide or gene has not been identified. While two relaxin-3-like genes were identified in the nearly completed chicken genome, no avian relaxin gene was found. As no other relaxin-like genes were found, the reported relaxin activity may be due to one of the relaxin-3-like genes.

The phylogeny of the relaxin-like peptide family indicates relaxin-3 is the ancestral relaxin, appearing prior to the divergence of teleosts. The finding of multiple relaxin-3-like genes in the fugu fish and zebrafish suggests multiple lineage-specific duplications of a single relaxin-3-like ancestor have occurred in fish [27]. However, the possibility the other mammal specific relaxin-like peptides emerged earlier before being lost in the teleost can not be excluded [27]. We find it more likely that these duplications, and the resulting multiple relaxin-3-like genes, are fish specific and due to genome wide duplications hypothesized to have occurred during fish evolution [43]. Phylogenetic analyses show multiple fish homologs of both the mammalian relaxin-3 and INSL5 genes, meaning that two relaxin-3-like genes existed prior to the genome duplication event proposed to have occurred in the teleost ancestor. The putative fish relaxin homolog was either, present in the teleost ancestor, duplicated and the second copy lost or emerged shortly after or, as a result of, the genome-wide duplication event.

While termed relaxin-3-like based on sequence similarity, phylogenetic analysis indicates that several non-mammalian sequences (OmRLX3, DrRLX3b, DrRLX3d, TrRLX3d, TrRLX3e and GgRLX3b) could be INSL5 homologs. None of the sequences found in the complete *X. tropicalis *genome were placed in this group, while there are members present in the more ancient fish lineage and the younger avian lineage. It is possible that this gene has either been lost, or remains unidentified, in the *X. tropicalis *genome. A sequence with similarity only to the B chain of relaxin-3 was also found, but a corresponding A chain match was not, however, there is a gap in the genome assembly upstream which might contain the missing domain. Future assemblies of the *Xenopus *genome should resolve this issue. These results suggest that INSL5 could have emerged during teleost evolution, far earlier than previously believed. Unlike the mammal-specific relaxin-like genes, which are clustered together (on chromosome 9 in the human and chromosome 19 in the mouse), *INSL5 *is localized independently (chromosome 1 in the human and chromosome 4 in the mouse). These findings are of particular interest in the analysis of INSL5, which is still functionally uncharacterised.

All the potential non-mammalian INSL5 homologs retain the relaxin-specific B chain [RxxxRxxI/V] motif, hence would be capable of interacting with the relaxin receptor, LGR7, and thus functionally classified as a relaxin. Recent studies have shown INSL5 is a high affinity ligand for GPCR142 but not GPCR135, LGR7 or LGR8 [19]. As the residues required for interaction with GPCR135 and GPCR142 are not known, it is unknown whether the non-mammalian INSL5 homologs would interact with GPCR142, GPCR135 and/or LGR7.

Phylogenetic results from this study suggest the presence of a relaxin homolog in fish and frogs, although not in the chicken. Relaxin sequences have previously been isolated and peptide sequenced from either the ovaries or testes of the edible frog [30], little skate (*Raja erinacea*) [44], spiny dogfish (*Squalus acanthias*) [45], Atlantic stingray (*Dasyatis sabina*) [46] and the sand tiger shark (*Odontaspis taurus*) [47]. While having high similarity with relaxin-3, these sequences are not relaxin-3 homologs (as the B chain of the stingray sequence is lacking the relaxin-specific motif, it is not a functional relaxin [46] and has not been considered further). Based on the expression of all these genes in reproductive organs such as the testes and ovaries, and the failure to find the *R. esculenta *gene expressed in the brain using northern blot analysis [30], we believe these to be among the first relaxin peptides with a reproductive function. Based on the similarity with relaxin-3 observed in these sequences, the ancestral relaxin homolog, and its new reproductive function, is likely to have emerged prior to the divergence of teleosts. A complete picture of relaxin-like peptides present in non-mammalian genomes will be invaluable in understanding the evolution of relaxin from neuropeptide to reproductive hormone.

The ancestral *RLN3 *gene is under very strong purifying selection, highlighting the importance of its highly conserved function, likely to be in the brain [2]. As high divergence is a hallmark of relaxin sequences, it is somewhat unsurprising that *RLN2 *is under only weak purifying selection. We suggest that this lack of selective pressure has contributed to the high sequence divergence seen between many relaxins (e.g. human and mouse) and the differences in relaxin's functions observed across mammals.

Information about the selective constraints placed upon these peptides, can provide valuable insight into the nature of interactions with their receptors. Based on selection pressures we can conclude that the interactions between relaxin-3 and GPCR135, INSL5 and GPCR142 are very specific, while the binding of relaxin to LGR7 is much looser. In this context the cross-reactivity seen between LGR7 and INSL3 or H1 relaxin, which are both similar to relaxin in sequence but especially in structure, is understandable, as is the lack of binding between GPCR135 and GPCR142 with any other relaxin-like peptide. Unexpectedly, synonymous and nonsynonymous substitution rate estimates for *RLN1 *and *INSL6 *show these to be under positive selection. Positive selection is often difficult to observe using pairwise comparisons that average over the whole length of a sequence, making these results even more striking. While pairwise comparisons failed to confirm positive selection was acting on *INSL4*, further statistical tests suggested that positive Darwinian selection acted on several sites in the *INSL4 *sequence after its emergence. Further analysis will be required to confirm these sites as important in the acquisition of a new receptor and a new function by INSL4, particularly in light of recent studies that question the reliability of ML methods to accurately detect positive selection acting on single sites [48-50]. We are encouraged that both branch-specific and site-specific ML models find positive selection to be acting on INSL4.

When the B and A domains of each gene were analyzed separately, further differences in selection pressures became apparent. The interaction between relaxin and its receptor has been thought to be primarily mediated through the B chain of the peptide [4], so the finding that selection pressures are stronger on the A chain of relaxin-1, INSL4 and INSL6 was unexpected. We also find it noteworthy that INSL4, INSL6 and relaxin-1 are the most recent members of the family to emerge and all appear to be under the effects of positive Darwinian selection. *INSL6 *emerged during mammalian development, *INSL4 *and *RLN1 *during primate evolution, they remain functionally uncharacterized and INSL4 and INSL6 are without known receptors. The low selection pressure on the B domain and the strong constraints placed on the A domain of INSL4 and INSL6 suggests that, unlike the B chain mediated interaction of relaxin and INSL3 with their receptors, the interaction of these peptides with their receptors could be dependant on the A chain instead. The low *d*_N_/*d*_S _rate observed for *INSL5 *indicates this peptide to be evolutionary stable and of functional importance. In particular the constraints placed on both A and B chains of INSL5 suggest a well-defined receptor interaction system, while the total absence of these constraints on either chain within relaxin-1 suggests the opposite, that perhaps this peptide is still evolving its function.

## Conclusions

We present here a phylogeny for the relaxin-like peptide family. Relaxin has long been used as an example of a gene that conflicts with the Darwinian theory of evolution [24,32-34]. However, we have shown that these can issues can be resolved when studied in the context of the rest of the relaxin-like peptide family, in particular the new, but likely ancestral relaxin, relaxin-3.

We have demonstrated that positive selection has been a driving force in the recent expansion of the relaxin-like peptide family during mammalian evolution. While strong purifying selection has maintained the structural core of these peptides by constraining the insulin superfamily cysteine motif, outside these residues, positive selection has acted after at least three gene duplication events (which generated INSL6, INSL4 and relaxin-1) to allow these new genes to acquire a new receptor and novel functions. Given the known roles of relaxin and INSL3 in reproduction (and the likely similar roles of INSL4 and INSL6 given the specificity of their expression in reproductive tissues) these findings correlate with a general trend towards rapid evolution in several reproduction associated genes [51-54]. We anticipate that further analysis of the coevolution of the relaxin-like peptides with their receptors will contribute much towards our understanding of the pleiotropic actions of this family as well as mechanisms involved in the evolution of peptide hormone systems.

## Methods

### Sequences and sequence similarity searches

Amino acid and nucleotide sequences of cloned relaxin-like peptide family members from the following species were obtained from GenBank [55]: human (*Homo sapiens*) H1 relaxin, H2 relaxin, H3 relaxin, INSL3, INSL4, INSL5, INSL6; mouse (*Mus musculus*) relaxin, relaxin-3, INSL3, INSL5, INSL6; rat (*Rattus norvegicus*) relaxin, relaxin-3, INSL3, INSL6; dog (Canis familiaris) relaxin; pig (*Sus scrofa*) relaxin, relaxin-3, INSL3; edible frog (*Rana esculenta*) relaxin and tammar wallaby (*Macropus eugenii*) relaxin (see table 2 for accession numbers). Five published relaxin-like sequences previously identified in the fugu fish (TrRLX3a-e) [27] and the zebrafish (DrRLX3a) [28] were also used. There are several partial relaxin-like peptide sequences available, however only sequences with corresponding nucleotide sequence data were utilized in this study.

Sequence similarity searches using TBLASTN [56] were conducted using the B and A chain sequences of each family member to identify other mammalian, vertebrate and invertebrate relaxin-like peptides. The following databases were searched: human, mouse, rat, dog, chimpanzee (*Pan troglodytes*), fugu fish, zebrafish, fruit fly (*Drosophila melanogaster*), mosquito (*Anopheles gambiae*), *Caenorhabditis elegans*, all yeast, all plant and all bacterial genomes at NCBI [57], *X. tropicalis *[58] and *C. intestinalis *[59], Expressed Sequence Tags (EST), Genome Survey Sequences (GSS), and High-Throughput Genomic Sequences (HTGS) databases [55]. While the classical cysteine motif of the insulin superfamily was used to distinguish sequences as members of this family, relaxin homologs were distinguished by the additional presence of the specific relaxin motif [RXXXRXXI/V] in the B chain of the derived peptide sequence.

### Multiple sequence alignment and phylogenetic analysis

Amino acid sequences were aligned using ClustalW [60] with default parameters. The alignments were edited to delete the C and signal peptide sequences, leaving only the B and A domains, which were further edited to minimize gaps and then concatenated. Human insulin was included as an outgroup.

Phylogenetic trees were constructed using maximum parsimony (MP): implemented in PHYLIP [61] using ProtPars, Neighbour-joining (NJ): implemented in PHYLIP using ProtDist and Neighbour and maximum likelihood (ML): implemented in Tree-Puzzle [62]. Data analyzed in PHYLIP was bootstrapped 1000 times using SeqBoot and consensus trees derived using Consense. Tree-Puzzle was run with a two-rate model of heterogeneity, the Dayhoff model of substitution and 50 000 puzzling steps. Trees were edited using TreeView [63].

### Reconciliation of gene and species trees

Gene trees of relaxin-like peptides were reconciled with a species tree using GeneTree [64]. Reconciled trees are an attempt to resolve incongruence between gene and species trees by predicting gene duplications and losses [64]. The species tree was based on a phylogeny of model organisms [65]. The reconciled tree was edited to minimize incongruence, primarily by reducing inferred duplications.

### Estimation of synonymous and nonsynonymous substitution rates

Pairwise nucleotide sequence alignments of human and chimpanzee, human and rhesus monkey (*Maca mulatta*) and human and mouse orthologs were constructed using ClustalW [60] and edited to limit alignments to the B and A domains only, which were then concatenated. Synonymous (*d*_S_) and nonsynonymous (*d*_N_) substitution rates were estimated using the methods of Yang and Nielsen [66] as implemented in yn00 in the PAML suite [67].

### Testing for positive selection

The following relaxin-1 and INSL4 nucleotide sequences were aligned using ClustalW: human H2 relaxin (X00948), INSL4 (L34838), chimpanzee(*Pan troglodytes*) relaxin-2 (Z27245), INSL4 (BK005152); gorilla (*Gorilla gorilla*) relaxin-2 (Z27228, Z27237); rhesus monkey relaxin (A34936), INSL4 (BK005251); bush baby (*Galago crassicaudatus*) relaxin (AF317625); baboon (*Papio hamadryas*) relaxin (Z27246, Z27224); camel (*Camelus dromedarius*) relaxin (AF254739); cat (*Felis catus*) relaxin (AF233688); dog (*Canis familiaris*) relaxin (AF233687); guinea pig (*Cavia porcellus*) relaxin (S85964); rabbit (*Oryctolagus cuniculus*) relaxin (S45940); hamster (*Mesocricetus auratus*) relaxin (S79879) and horse (*Equus caballus*) relaxin (AB000201). The alignment was edited as described previously. A ML tree was constructed from this alignment using TreePuzzle [62] and the method of Yang and co-workers [31] was used to test for positive selection in the INSL4 branch. Using Codeml from the PAML suite, several models were fitted to the data. The branch specific models, One-ratio (R1) and Two-ratios (R2) were used to detect lineage-specific changes in selective pressure. The site specific models, Neutral (M1), Selection (M2), Discrete (M3) with 2 and 3 site classes, Beta (M7) and Beta&ω (M8), were also used to test for individual residues under positive selection. The branch-site models A and B were used to detect positive selection in a subset of sites in a specified branch. Likelihood ratio tests (LRT) were used to assess their goodness of fit, by comparing a model that does allow for dN/dS > 1 against a model that does not (i.e. a null model). Therefore, the branch specific LRT was R2 vs. R1. The site specific LRTs were M3, M2 and M8 against their respective null models, M0, M1 and M7. The branch-site models A and B were tested against M1 and M3 with k = 2 site classes respectively. Positively selected sites with a posterior probability of *P*_(ω>1) _>0.90 were listed.

## Authors' contributions

TW performed all sequence and phylogenetic analysis and drafted the manuscript, TS participated in phylogenetic analysis, design and coordination of study, GWT participated in the design of the study, and RADB participated in phylogenetic analysis, conceived of the study and participated in its design and coordination.

## Supplementary Material

Additional File 1 **Phylogeny of cluster A- relaxin-3 and INSL5. **Phylogeny of Cluster A constructed from a ClustalW alignment of the B and A domain amino acid sequences from relaxin 3 and INSL5 peptides. Consensus tree generated from MP (Protpars in PHYLIP), ML (TreePuzzle) and NJ (Neighbour in PHYLIP) methods and edited in Treeview to minimize species tree incongruence. Human insulin was used as an outgroup. Where possible, confidence values are shown at branches: * >50%, ** >75%, all other branches are inferred. Hsa = *Homo sapiens*, Pt = *Pan troglodytes*, Mm = *Mus musculus*, Rn = *Rattus norvegicus*, Cf = *Canis familiaris*, Ss = *Sus scrofa*, Xt = *Xenopus tropicalis*, Dr = *Danio rerio*, Tr = *Takifugu rubripes*, Gg = *Gallus gallus*, Om = *Oncorhynchus mykiss*. Click here for file

Additional File 2 **Phylogeny of cluster B- relaxin-1, 2, INSL3, INSL4 and INSL6. **Consensus phylogeny of Cluster B constructed from a ClustalW alignment of the B and A domain amino acid sequences from relaxin 1, 2, INSL3, INSL4, INSL6 peptides. Consensus tree generated from MP (Protpars in PHYLIP), ML (TreePuzzle) and NJ (Neighbour in PHYLIP) methods and edited in Treeview to minimize species tree incongruence. Human insulin was used as an outgroup. Where possible, confidence values are shown at branches: * >50%, ** >75%, all other branches are inferred. Hsa = *Homo sapiens*, Pt = *Pan troglodytes*, Mmul = *Maca mulatta*, Mm = *Mus musculus*, Rn = *Rattus norvegicus*, Cf = *Canis familiaris*, Ss = *Sus scrofa*, Re = *Rana esculenta*, Me = *Macropus eugenii*, Xl = *Xenopus laevis*, Xt = *Xenopus tropicalis*, Dr = *Danio rerio*, Tr = *Takifugu rubripes*. Click here for file
