# Supplementary material for: Evolution of the relaxin-like peptide family
Source: BMC Evol Biol. 2005 Feb 12;5:14. doi: 10.1186/1471-2148-5-14 (PMC551602; doi:10.1186/1471-2148-5-14)
Supplement: Additional File 1 — Phylogeny of cluster A- relaxin-3 and INSL5. Phylogeny of Cluster A constructed from a ClustalW alignment of the B and A domain amino acid sequences from relaxin 3 and INSL5 peptides. Consensus tree generated from MP (Protpars in PHYLIP), ML (TreePuzzle) and NJ (Neighbour in PHYLIP) methods and edited in Treeview to minimize species tree incongruence. Human insulin was used as an outgroup. Where possible, confidence values are shown at branches: * >50%, ** >75%, all other branches are inferred. Hsa = Homo sapiens, Pt = Pan troglodytes, Mm = Mus musculus, Rn = Rattus norvegicus, Cf = Canis familiaris, Ss = Sus scrofa, Xt = Xenopus tropicalis, Dr = Danio rerio, Tr = Takifugu rubripes, Gg = Gallus gallus, Om = Oncorhynchus mykiss. [file 1471-2148-5-14-S1.doc]

Additional figure 1

**

*

**

*

**

*

**

**

**

**

**

**

*

*
