# Supplementary material for: Evolution of the relaxin-like peptide family
Source: BMC Evol Biol. 2005 Feb 12;5:14. doi: 10.1186/1471-2148-5-14 (PMC551602; doi:10.1186/1471-2148-5-14)
Supplement: Additional File 2 — Phylogeny of cluster B- relaxin-1, 2, INSL3, INSL4 and INSL6. Consensus phylogeny of Cluster B constructed from a ClustalW alignment of the B and A domain amino acid sequences from relaxin 1, 2, INSL3, INSL4, INSL6 peptides. Consensus tree generated from MP (Protpars in PHYLIP), ML (TreePuzzle) and NJ (Neighbour in PHYLIP) methods and edited in Treeview to minimize species tree incongruence. Human insulin was used as an outgroup. Where possible, confidence values are shown at branches: * >50%, ** >75%, all other branches are inferred. Hsa = Homo sapiens, Pt = Pan troglodytes, Mmul = Maca mulatta, Mm = Mus musculus, Rn = Rattus norvegicus, Cf = Canis familiaris, Ss = Sus scrofa, Re = Rana esculenta, Me = Macropus eugenii, Xl = Xenopus laevis, Xt = Xenopus tropicalis, Dr = Danio rerio, Tr = Takifugu rubripes. [file 1471-2148-5-14-S2.doc]

Additional figure 2

**

**

**

**

*

*

**

**

**

**

**

**

*

*

*

*
